# Weak evidence of bright light effects on human LH and FSH

**DOI:** 10.1186/1740-3391-8-5

**Published:** 2010-05-11

**Authors:** Daniel F Kripke, Jeffrey A Elliott, Shawn D Youngstedt, Barbara L Parry, Richard L Hauger, Katharine M Rex

**Affiliations:** 1 Department of Psychiatry, University of California, San Diego, La Jolla, California 92093, USA; 2 Department of Exercise Science, University of South Carolina, Columbia, South Carolina, USA; 3 San Diego VA Healthcare System, Psychiatry Service, San Diego, CA 92161, USA

## Abstract

**Background:**

Most mammals are seasonal breeders whose gonads grow to anticipate reproduction in the spring and summer. As day length increases, secretion increases for two gonadotropins, luteinizing hormone (LH) and follicle stimulating hormone (FSH). This response is largely controlled by light. Light effects on gonadotropins are mediated through effects on the suprachiasmatic nucleus and responses of the circadian system. There is some evidence that seasonal breeding in humans is regulated by similar mechanisms, and that light stimulates LH secretion, but primate responses seem complex.

**Methods:**

To gain further information on effects of bright light on LH and FSH secretion in humans, we analyzed urine samples collected in three experiments conducted for other goals. First, volunteers ages 18-30 years and 60-75 commenced an ultra-short 90-min sleep-wake cycle, during which they were exposed to 3000 lux light for 3 hours at balanced times of day, repeated for 3 days. Urine samples were assayed to explore any LH phase response curve. Second, depressed participants 60-79 years of age were treated with bright light or dim placebo light for 28 days, with measurements of urinary LH and FSH before and after treatment. Third, women of ages 20-45 years with premenstrual dysphoric disorder (PMDD) were treated to one 3-hour exposure of morning light, measuring LH and FSH in urine before and after the treatments.

**Results:**

Two of the three studies showed significant increases in LH after light treatment, and FSH also tended to increase, but there were no significant contrasts with parallel placebo treatments and no significant time-of-day treatment effects.

**Conclusions:**

These results gave some support for the hypothesis that bright light may augment LH secretion. Longer-duration studies may be needed to clarify the effects of light on human LH and FSH.

## Background

Several generations of scientists have studied photoperiodism extensively throughout the biological world. One of the most dramatic photoperiodic responses in birds is a massive increase in luteinizing hormone (LH) appearing within a few hours of stimulatory light exposure at a critical time of night [1]. Likewise, in small mammals, light at critical times of night produces large increases in LH and follicle stimulating hormone (FSH), resulting in growth of gonads [2-4]. These increases in gonadotropins prepare animals for seasonal breeding [5]. There is a preponderance of evidence that light at night functions to decrease the duration of melatonin secretion, causing the nights to be interpreted as shorter (complementing a longer summer-like day) [6-8]. However, some evidence for critical time intervals for light (or melatonin) sensitivity at night suggests a more complex mechanism [9,10]. Moreover, in hamsters, light may stimulate LH at times outside the normal interval of melatonin secretion [4]. In mammals, neurobiologic responses to light are intrinsically bound to the circadian timing system [4,5,11].

The relationships between melatonin peak duration and LH stimulation are species-specific and may be somewhat different in large mammals, whose gestation period may require autumn-winter breeding for births to occur in the spring. LH is regulated photoperiodically in sheep, but short days (longer nights) stimulate LH in order to implement autumn-winter breeding, which results in spring births [12]. Nonhuman primates are also photoperiodic breeders, but the situation is complex, as some primates breed (and show LH peaks) in autumn or winter [13]. In female rhesus monkeys, light increased multiunit activity of hypothalamic neurons (which were presumably those GnRH neurons which stimulate LH release) [14].

Although seasonal elevations of LH may occur at various times of year, depending on the seasonal breeding adaptation, prolactin elevations occur in summer in both summer-breeding and most winter-breeding mammals [15]. Humans may be an exception [16].

Much progress has been made in understanding the mechanisms of mammalian photoperiodism. The light stimulus is probably transmitted from retinal neurons containing the photopigment melanopsin and supplying the axons of the retino-hypothalamic tract [17-20], which synapse within the suprachiasmatic nucleus (SCN), releasing neurotransmitters PACAP, glutamate, and perhaps acetylcholine [21]. Accessory pathways to the SCN may be involved, including pathways from the serotonin-releasing cells of the midbrain and the intergeniculate leaflet cells releasing NPY and GABA. SCN cell-surface receptors regulate the transcription of circadian clock genes through complex pathways [20,22]. The SCN appears to be a crucial center of photoperiodic control [23]. The timing of dawn and dusk influences SCN functional circadian organization, apparently through differential entrainment effects on morning and evening circadian oscillator components [24-26]. It is possible that separate evening and morning components are represented in the molecular circadian clock, e.g., by PER1, PER2, and the cryptochromes, the circadian phase relationships of which, somehow mediate photoperiodism [27-30]. Thus, the timing of light exposure may influence the dynamics of the SCN molecular circadian clock. An important output of the SCN controls the nocturnal release of melatonin from the pineal. The secretion of melatonin is longer in long nights (short days), but may be abbreviated by brief light exposures during the night [6]. There is much evidence that the pineal and melatonin are necessary for the inhibition of gonadal function produced by short days. It appears that melatonin effects on hypothalamic synthesis of active T3 and reverse T3 may mediate some of these responses [31,32].

Regulation of LH and FSH is responsive to the circadian timing of light, to the absolute photoperiod, and also to the history of change in photoperiod [4,33]. Pituitary release of LH is largely governed by the frequency of pulsatile releases of gonadotropin releasing hormone (GnRH) into the portal circulation [34]. Part of the response involves sensitivity to negative feedback of gonadal hormones (such as estrogen) upon GnRH cells [35]. There is considerable interspecies variation in the anatomy and connections of GnRH cells. GnRH cells tend to be anterior to the SCN in rodents, but in humans, GnRH is released mainly from cells of the arcuate nucleus posterior to the SCN. The neurophysiologic pathways controlling GnRH are not as clear as with prolactin. In sheep, melatonin seems to influence neurons in the posterior or median hypothalamus which use several neurotransmitters to modulate the GnRH neurons [12,35]. There are also direct synaptic connections to GnRH neurons from SCN cells, which might transmit the photoperiodic regulatory message [14,36,37]. A possible SCN neurotransmitter or diffusable messenger is prokineticin 2, for which a dense receptor supply is found in the arcuate [38]. Kisspeptin is also thought to be released by the SCN [39-41]. The exact mechanisms by which day length may be transduced to control arcuate nucleus pulsatile GnRH release remain somewhat a mystery.

FSH pulsatile release is mediated to some extent by GnRH release. However, there is also a chemically-related peptide more specific for releasing FSH, called FSHRF [42]. The cells releasing FSHRF in the rat have somewhat distinct anatomic distributions compared to the GnRH cells.

There is intriguing evidence that humans are photoperiodic, but the data are not entirely consistent. Human melatonin secretion has a longer duration in winter than summer [13,43-46]. Human reproduction varies somewhat by season; moreover, seasonal effects interact with latitude [47,48]. There are two peaks of births per year in some human population data sets. In hot climates, air temperature may be a factor [49]. Regarding the reproductive hormones, one study found a small June LH peak in young men from the west coast of America [50]. Similarly, in elderly Romanian men and women, LH was higher in spring and summer [51]. In contrast, February-March and August-September LH peaks were found in male and female Italian children ages 6-10 years [52]. In Finnish women, 6-hour midfollicular LH was greater in December than April-May [53]. Some discrepancies between studies may be due to failure to control adequately for circadian effects in hormones which undergo both circadian and seasonal modulation.

In a small study of 11 healthy young men, our laboratory found that 1000 lux bright light from 0500-0600 in the morning for 5 mornings could increase daily LH production as much as 65% [54]. This dramatic augmentation of LH production by bright light needed replication. It also raised many additional questions: What would happen with light treatment at other times of day? What would happen with longer durations of daily treatment? What would happen after several weeks of treatment? Will similar responses occur in older men who may be more in need of LH-testosterone augmentation? Would similar responses occur in women, in whom there is preliminary evidence that light may augment LH, FSH, and ovulation [55]? Would the effect in women depend upon menstrual cycle phase or upon menopause?

To seek data relevant to these questions, we assayed LH and FSH in samples from three human studies undertaken for other goals.

## Methods

### Study 1, PRCs

A series of experiments were performed to examine light circadian phase-response curves (PRCs), contrasting adult women and men of young and older ages [56]. Baseline circadian phase was assessed by monitoring subjects for 30-48 hours in the laboratory while they underwent an ultra-short 90-min sleep-wake cycle, consisting of a 30 min. lights-out-sleep period followed by a 60 min. lights-on-wake interval in 50 lux. Urine samples collected by the participants were frozen every 90 min. (2 ml) for later assays, to measure urinary aMT6s (6-sulfatoxymelatonin), the primary metabolite of melatonin. After aMT6s was assayed, the refrozen samples were subsequently rethawed for urinary LH assays.

Using stratified randomization, subjects were assigned to receive 3000 lux light treatment for 3 hours on each of 3 consecutive days at one of 8 times of day, while continuing the 90-min ultra-short sleep-wake cycle. Because of variability in baseline circadian phase adjustment, light treatments were effectively administered randomly throughout the 360-degree circadian cycle. Then a 30-hour follow-up assessment was made with further urine collections every 90 minutes.

LH was assayed in rethawed baseline and follow-up samples with the DSL-10-460 Active^R ^LH Elisa, an enzymatically amplified "one-step" sandwich-type immunoassay (Diagnostic System Laboratories, Inc., Webster, TX.) Standards (0 to 100 mIU/ml), controls and unknowns were incubated with an anti-LH antibody in micro plate wells coated with another anti-LH antibody. After incubating and washing, the wells were incubated with tetramethylbenzidine (TMB) substrate and the timed reaction stopped with an acidic solution. Finally, enzymatic turnover of the substrate was quantified by dual wavelength (450 and 630 nm) absorbance measurement in a micro plate reader. With the above protocol, the DSL LH EIA displays a sensitivity of 0.1 mIU/ml with intra-assay and inter-assay coefficients of variation ranging with mean dose (2.8 to 69.2) from 5.3 to 7.6%. Urine samples were typically measured by diluting 1:1 with zero standard. The concentration of LH or FSH was multiplied by the urine volume per time to obtain excretion during each sampling interval expressed as mIU/h.

By assaying LH in the same urine specimens in which melatonin had been measured (aliquots of which remained frozen at -70^ο^C), we produced LH-response curves resembling light phase-response curves [56], to indicate increases or decreases in LH following light treatment at contrasting circadian phases. The LH response was the 24-hour excretion rate for LH during the follow-up divided by the 24-hour excretion rate during the baseline. Because these responses formed a highly skewed distribution, the responses were then normalized as the Log_10 _of this ratio, the response index. These logged normalized responses formed a normal distribution. The circadian times of optimal response were sought. A dead zone for LH stimulation was predicted near mid-day, similar to that observed in light responses [56]. Such a stimulation dead zone might incidentally serve the function of a control-placebo condition. Responses of older and younger adults were compared.

Advantages of this design were that the responses of subjects ages 18-30 years and 60-75 years could be directly contrasted in the same protocol, and LH responses to light could be observed at all circadian times. A limitation was that although an attempt was made to study all women ages 18-30 years during the follicular menstrual phase, there were various operational scheduling problems. Unfortunately, the menstrual phases were not physiologically verified, so studies with better control of menstrual phase are needed for young women.

### Study 2, Light treatment of depression

Our group tested the value of bright light treatment for depression in a controlled clinical trial, recruiting participants 60-79 years of age [57]. Subjects were selected for a Geriatric Depression Scale [58] score of at least 11 at baseline, which indicated probable major depressive disorder. An actual research diagnosis of major depression was not required, because many older people suffer substantial handicaps from depression without meeting formal diagnostic criteria for major depression [59]. Participants were studied in their own homes and community, while they continued with any ongoing treatment, which sometimes included stable utilizations of antidepressant drugs and psychotherapy. Light was tested as an augmenting therapy along with antidepressants and psychotherapy. Usage of drugs which distort melatonin secretion was an exclusion criterion, in order not to invalidate aMT6s measurements. At the end of a baseline week during which all subjects received mid-day placebo treatment, subjects collected all fractional urine specimens for 24 hours. Based on actigraphy, subjects were phase typed to predict whether they were somewhat phase-advanced or phase-delayed, and therefore might best respond to early morning light (administered immediately after awakening), mid-day light (no phase adjustment desired), or evening light (administered from 1-2 hours before bedtime). After the phase-typing to predetermine treatment timing, using structured randomization, subjects were then randomly assigned either to bright light treatment (10,000 lux for 1 hour) or dim red (<10 lux for 1 hour) placebo light treatment, to continue for 28 days. Wrist actigraphic monitoring, sleep logs, and self-ratings were carried out throughout the study. At the end of the 28 days, urine collections were repeated for 24 hours, as well as mood ratings. LH was assayed as described above, using the DSL-10-460 Active^R ^LH Elisa. Since we only saw tentative actigraphic and mood evidence of light effects in the morning-light group, we limited our LH assays to that group.

### Study 3, PMDD

The bright light responses of menstruating women were studied, contrasting those with and without premenstrual dysphoric disorder (PMDD), as an extension of previous work [60]. The women with PMDD who were selected for this study regularly became depressed during the luteal menstrual phase. In pilot studies, adult women with PMDD exhibited an abnormal phase shift to early morning light, suggesting some disorder of the circadian system. This study contrasted the phase-shifting responses to early-night and early-morning bright light in women with DSM-IV PMDD with the responses of normal controls. The age range was 20-45 years. First, the women underwent extensive diagnostic evaluations and preliminary Clinical Research Center admissions to determine their dim light melatonin onset (DLMO) in the follicular and luteal menstrual phases. DLMO is that time of evening when the onset of melatonin secretion occurs, an excellent circadian timing marker, which is used to insure that each subject can be exposed to bright light at standardized phases of the circadian rhythm, even though circadian rhythms differ in phase-timing from one subject to another. Then, in separate months, each woman underwent two Clinical Research Center admissions to test bright light responses, once in the follicular menstrual phase (8 ± 2 days after the onset of menses) and once in the luteal menstrual phase (2-4 days before the date of next expected menses, over a month later).

Each of these Clinical Research Center admissions included a baseline urine-sampling night, a bright light treatment night, and an additional follow-up night for urine sampling to determine hormonal results of light treatment. Apart from the treatment, subjects were maintained in dim light or darkness. Fractional urine specimens were measured and sampled both during the baseline (6 PM to 11 AM) and during the follow-up (6 PM to 11 AM). Part of each group (PMDD and controls) received bright 6000 lux light for 3 hours early in the night, starting 3 hours after the DLMO (approximately 24:00-0300). Also, the other half of each group (PMDD and controls) received similar bright light early in the morning starting 8 hours after the DLMO (approximately 05:00-08:00). Blood and urine samples were assayed for melatonin, to determine the phase of the circadian system, which is indicated well by the nocturnal rise and morning fall of melatonin. The bright light administered starting 3 hours after the DLMO was expected to be phase-delaying. The bright light starting 8 hours after the DLMO was expected to be phase-advancing in controls, but possibly not among PMDD patients.

Urinary LH and FSH data were analyzed in SPSS 12.0 with a multivariate repeated-measures general linear model. The main within-subjects factor was the baseline to after-light-treatment change within each CRC admission. The secondary within-subjects factor was the follicular phase and luteal phase repetitions of the light treatment protocol. A between-subjects factor was light treatment in the morning or evening. Since hormonal results were highly skewed, the log_10 _transformations of excretion were utilized.

## Results

### Study 1, PRCs

The participants were 25 young women with a mean age of 24.0 years (range 18-31), 17 young men with a mean age of 23.6 years (range 18-30), 28 older women with a mean age of 66.2 years (range 60-74), and 28 older men with a mean age of 67.8 years (range 59-75).

The mean 24-hour LH excretion rate before light treatment was 597 mIU/h. The young females secreted more than 4 times as much LH as young males, but older participants of either gender were intermediate and about equal (ANOVA gender effect P = 0.028, age effect NS, age/gender interaction P = 0.03). LH excretion was more than twice as high in the April-June quarter as in January-March (with July-December intermediate), but the ANOVA for season effect controlled for age and gender was not significant, nor did the LH response after light treatment differ significantly by season of the year.

**Figure 1:**
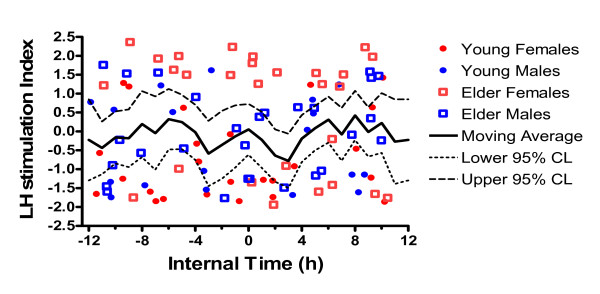
**Change in LH in relation to time of light exposure**. Change in urinary LH excretion (mIU/ml) is expressed as the LH stimulation index. This is a change index calculated by computing the ratio of the post-treatment LH excretion rate divided by the baseline (pre stimulus) rate, and then taking the log (base 10) of this fraction. In this way, a decrease producing a ratio of < 1.0 gives a negative log value. Each point is plotted relative to the internal circadian time of the midpoint of the 3 h light stimulus. Zero h represents circadian midnight, defined as 3.52 h prior to the time of the aMT6s acrophase in the pre-stimulus phase assessment. The key to symbols identifies the points by age and gender of the participants, N = 98. The black line presents a 3-hour moving average of the points, with dotted lines showing 95% confidence intervals of the mean.

**Figure 2:**
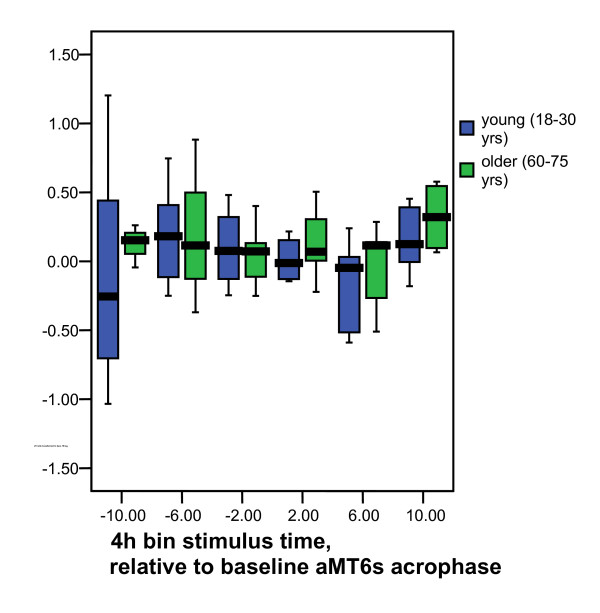
**LH responses grouped by age group and times of light stimulus**. The log LH response index (as described for Figure 1) is shown separately for young and older subjects versus the time of stimulus (in reference to the acrophase-peak of 6-sulfatoxymelatonin). Thick horizontal lines are medians. Blue and green boxes are interquartile ranges. The thin bars represent the range for each time bin.

After treatment, mean excretion was 719 mIU/ml. The gender effect persisted post treatment (P < 0.04), but the interaction with age did not. The mean ratio of excretion after/before treatment was 1.62 ± SE 0.19. The increase in log_10_(LH) post treatment, controlled for age group and gender, was significant (P = 0.03). Thus, the Log_10 _mean excretion index was .063. The timing groups did not differ significantly by log_10_(LH response), which averaged close to zero for all groups, independent of time of light stimulation. As shown in Figures 1 and 2, the moving average responses were close to 0 at all times of day. Neither for the 98 participants as a whole nor for any of the age-gender subgroups (not shown) was there a consistently elevated response at a particular time of day.

### Study 2, Light treatment of depression

Effects of the light treatment on mood and sleep, which were quite minimal and equivocal, were reported elsewhere [57]. There were 7 subjects assigned to bright morning light and 7 assigned to dim morning light who had provided adequate urine samples which could be assayed satisfactorily.

The Spearman rank-order correlation of LH and FSH excretion before light treatment was R_s _= 0.62 (P < 0.02) among 14 participants. The correlation of LH excretion before and after light (dim control or bright) was R_s _= 0.72 among 14 participants (P = 0.004). The correlation of FSH excretion before and after light was only R_s _= 0.34 (NS). Excretion per hour of LH and FSH did not differ significantly between bright-light and dim-light treated groups, either before or after treatment. Both LH and FSH tended to increase after treatment, but the trend was for a greater LH increase after dim light and a greater FSH increase after bright light (NS).

### Study 3, PMDD

Complete measurements of urinary LH and FSH before and after light treatment in the both the follicular and luteal phases were available for only 4 women given morning light and 2 women given evening light.

The multivariate contrast of hormone measures before and after the bright light stimulation was significant (P = 0.044). Log_10_(LH) excretion increased from 1.047 on the baseline night to 1.264 after the bright light treatment (univariate P = 0.027). Log_10_(FSH) excretion increased from 0.030 on the baseline night to 0.196 on the night after the bright light treatment (univariate P = 0.125). The follicular vs. luteal phase hormonal excretion contrasts were not significant, nor were the morning vs. evening light treatment contrasts, but the interaction of the pre-post hormonal excretion contrast with time of treatment was significant (P = 0.008). For LH, the increase after morning light treatment was consistently higher than with evening light in both the follicular and luteal phases. For FSH, the increase with morning light treatment was higher than with evening light in the follicular phase, but in the luteal phase, FSH excretion was extremely reduced, and the very small increase was greater with evening than with morning light. There were no other significant interactions.

## Discussion

To summarize, assays of urinary LH and FSH before and after bright light treatments gave weak and somewhat equivocal support to the hypothesis that bright light stimulates these gonadotropic hormones. Light stimulation of LH and FSH would be potentially useful, if a robust effect could be more clearly demonstrated. In children, regulation of light exposures might influence puberty and menarche. In men, an increase in testosterone production might lead to increased fertility in some younger men, and palliation of loss of libido, erectile dysfunction, and muscle wasting among older men. In women, light stimulation might promote fertility as well as regularize the menstrual cycle [55,61]. In both genders, bright light augmentation of gonadotropins might be one aspect of the antidepressant benefits [62-64].

In the first and third studies, LH was significantly increased after bright light treatment, but in neither case was a significant contrast with any parallel control treatment demonstrated. Therefore, we cannot exclude that the increases in LH were due to various placebo and nonspecific experimental effects, as well as the passage of time. For example, suggestion and hope often produce positive placebo responses in a wide variety of trials. If a clinical trial commenced with subjects in a heightened state of anxiety or depression, spontaneous remission might lead to endocrine changes such as enhancements of LH and FSH production. In a separate study, our group demonstrated a significant increase in FSH after early morning stimulations with bright green light, as compared to dim light placebo, but this effect was not very robust [65]. We are left with tantalizingly suggestive evidence that bright light stimulates LH and FSH in human subjects, but there is a need for much more convincing evidence.

These studies had a number of limitations. In the first study, the light stimuli were administered for only 3 days at a level of only 3000 lux, whereas, a longer duration of brighter stimulation might have produced a greater gonadotropic response. In the second study, though 10,000 lux bright light stimuli were randomized for 4 weeks, we were uncertain of the compliance of these very depressed and elderly study participants, particularly since there was only weak evidence for circadian phase-shifting effect in the experimental groups and no persuasive evidence of a favorable mood effect. Only data from the morning light treatments were analyzed because of lack of evidence that the evening treatments had any physiologic or mood effects. In the third study, the numbers of subjects were extremely small, and the light stimulation was given only on a single day, which may have been insufficient to produce a large effect. A light stimulus intended to produce a phase shift may not be optimal for stimulating LH and FSH. Though we were able to obtain data from these studies which had been organized for other goals, the numbers of participants yielding usable data was unexpectedly small in the second and third studies. There is also concern that the several years that the urine samples were frozen, and the freezing and thawing processes, might have introduced inaccuracy into the assays.

## Conclusions

Larger studies organized specifically to measure bright light effects on gonadotropins may be needed to verify the potential of bright light regulation for the reproductive endocrine system. Longer durations of exposure should be tested.

## Competing interests

The authors declare that they have no competing interests.

## Authors' contributions

DFK participated in design, acquisition of data, interpretation and statistics, and manuscript preparation. JAE provided intellectual background, performed the assays, and contributed to design, interpretation, statistics, and manuscript preparation. SDY contributed to design, performance, analysis, and interpretation of the first study. BLP designed and performed the third study and contributed to manuscript preparation. RLH consulted on the assays, provided laboratory facilities, and helped edit the manuscript. KMR managed human subjects consents and reimbursement, helped perform the first study, and contributed to manuscript preparation. All authors read and approved the final manuscript.
